# Patient Satisfaction and Quality of Life After Mastectomy at King Abdulaziz Medical City, Jeddah

**DOI:** 10.7759/cureus.51029

**Published:** 2023-12-24

**Authors:** Mussab A Barkar, Zaher Mikwar, Adil A Khalid, Ali A Mohammedamin, Abdulrahman H Aloufi, Abdulmajeed A Abualhamail, Hamad A Alghashim

**Affiliations:** 1 College of Medicine, King Saud Bin Abdulaziz University for Health Sciences, Jeddah, SAU; 2 College of Medicine, King Abdullah International Medical Research Center, Jeddah, SAU; 3 Department of General Surgery, Department of Surgery, King Abdulaziz Medical City, Jeddah, SAU

**Keywords:** saudi arabia, patients satisfaction, quality of life, mastectomy, breast cancer

## Abstract

Background

Overall well-being after surgical intervention is one of the most important aspects of assessing quality of life (QOL), yet it is not well explored in the literature. In this paper, it was necessary to involve the patient's perspective of the nature of their QOL. The burden of being diagnosed with breast cancer is an adaptation to a new lifestyle, having to deal with disease stigma, interpersonal relations problems, and being limited to specific clothing. This can be very challenging for patients. This study aims to identify which patient group, based on their treatment regimen, exhibits higher levels of satisfaction and dissatisfaction compared to other groups.

Methods

A retrospective, cross-sectional study analyzing the QOL among female breast cancer patients who underwent mastectomy, with or without breast reconstruction, in King Abdulaziz Medical City, Jeddah, between 2009 and 2022. Patients' demographics and phone numbers were obtained from each patient's medical record file in our hospital. Phone call-based interviews were conducted to contact patients to assess their QOL, satisfaction, and regrets after surgery. We excluded patients who do not speak Arabic, are illiterate, have memory disorders, patients who underwent lumpectomy or palliative mastectomy, patients with metastatic stage 4 cancer at the time of diagnosis, patients who are males, and patients who passed away.

Results

A total of 2,309 patients were screened during the period aforementioned; a total of 346 patients met our inclusion criteria. All of whom are female participants with a current mean age of 52.3 ± 11.5 years. There were 301 (86.99%) participants reported being satisfied, while only 45 (13.01%) participants reported being unsatisfied with surgery outcomes. Although the majority of participants were satisfied after mastectomy, many of them still struggled with psychological, social, and/or emotional challenges. These challenges can have a significant impact on a patient's overall well-being and QOL and must be addressed to provide patients with the highest quality of care possible.

Conclusion

The study findings highlight the significant impact of mastectomy on patients' lives. It is important to consider individual patient experiences and circumstances when evaluating treatment outcomes and patient satisfaction. We observed that patient satisfaction may vary depending on several factors, including patients' baseline satisfaction. Those factors may be psychological, such as body image issues, low self-esteem, the feeling of losing a body part, and fear of recurrence or metastasis. Other factors may be postoperative-related complications, including lymphedema, redundant skin, chronic pain, and operation scar. Additionally, factors may be socially related, such as loss of confidence, social withdrawal, embarrassment, inability to buy prostheses, being limited to specific clothes, and occupational impact.

## Introduction

Breast cancer (BC) has become the most commonly diagnosed type of cancer globally, according to a paper published by the Department of Surgery at Oxford University Hospitals [1]. It is the second cause of death among women in the United States [2]. Additionally, the leading cause of death among women in Saudi Arabia during the period of 2010-2019 was BC [3]. Healthcare workers and institutions have aimed to provide the best possible medical care for all patients. With the advancement of medicine and healthcare, new protocols and techniques were utilized to detect BC as soon as possible to prevent metastasis, increase the five-year survival rate, and preserve the aesthetic appearance [4]. A study was conducted in Australia using the BREAST-Q questionnaire to measure satisfaction post-mastectomy [5]. The results found that satisfaction was significantly higher in the reconstructed group in comparison to the non-reconstructed group, in which the main reason for seeking the reconstruction was to improve their self-image [6]. Meanwhile, another study from China showed that there was no convincing evidence for immediate breast reconstruction (BR) to have any benefit over mastectomy alone [7]. Moreover, the literature states that psychological well-being is a persisting problem in such patients. Patients' lives are affected by different aspects, including personal, institutional, and familial challenges [8,9]. A systematic review of depression among females with BC was conducted in 2018 to compare three surgical treatment options: total mastectomy (TM), breast conservative surgery (BCS), and BR. The results proved that there was no statistical significance among the three surgery approaches concerned with the occurrence of depressive symptoms. Women who are diagnosed with any stage of BC face various challenges and are psychologically vulnerable, especially after surgery. Breast surgery can affect BC patients majorly whether physically, emotionally, and/or socially [9]. Subjectively, the societal perception could be negative toward BC patients who underwent a mastectomy. Their satisfaction may vary depending on patient-related factors and/or surgery-related outcomes.

The purpose of this study was to measure the satisfaction of female BC patients who underwent a mastectomy, with or without reconstruction, in King Abdulaziz Medical City between the years 2009 and 2022. The investigators theorize that the satisfaction of a BC patient is impacted post-mastectomy. The specific aims were to investigate the satisfaction of mastectomy patients and analyze the reason for their satisfaction or the lack thereof and the impact of mastectomy on the lives of these patients physically, mentally, and socially.

## Materials and methods

Subjects and experimental design

This is a retrospective, cross-sectional study that analyzes the quality of life (QOL) among female breast cancer patients who underwent mastectomy whether unilateral or bilateral mastectomy, with or without breast reconstruction surgery, at our institute, King Abdulaziz Medical City, Jeddah, between 2009 and 2022.

Data collection and measurements

Phone call-based interviews were conducted to contact patients via female data collectors to minimize patient discomfort throughout the interview. Data collectors have verbally administered a questionnaire, asking patients about their experience and opinions about the surgery outcomes and results. There was a script for data collectors to follow while asking the patients. The script was well revised, considering cultural, social, and privacy aspects. The script can be found in the appendices section. The aim is to analyze the QOL of breast cancer patients by analyzing the satisfaction and regrets of patients about the surgery outcomes.

We have not used numerical scaling questionnaires for the patient’s state of satisfaction as in previous studies; instead, we developed our own questionnaire, which was validated through the face and content validity by experts in the surgical field and qualified language experts to ensure the clearance and competence of this questionnaire before administering it to patients. This was largely to measure the perspectival nature of patients' QOL. The aspects of patients' lives we have shed light on were the emotional, social, and functional status of patients. The aim is to raise awareness about the interpersonal challenges that patients experience throughout their journey from diagnosis to treatment of BC.

**Figure 1:**
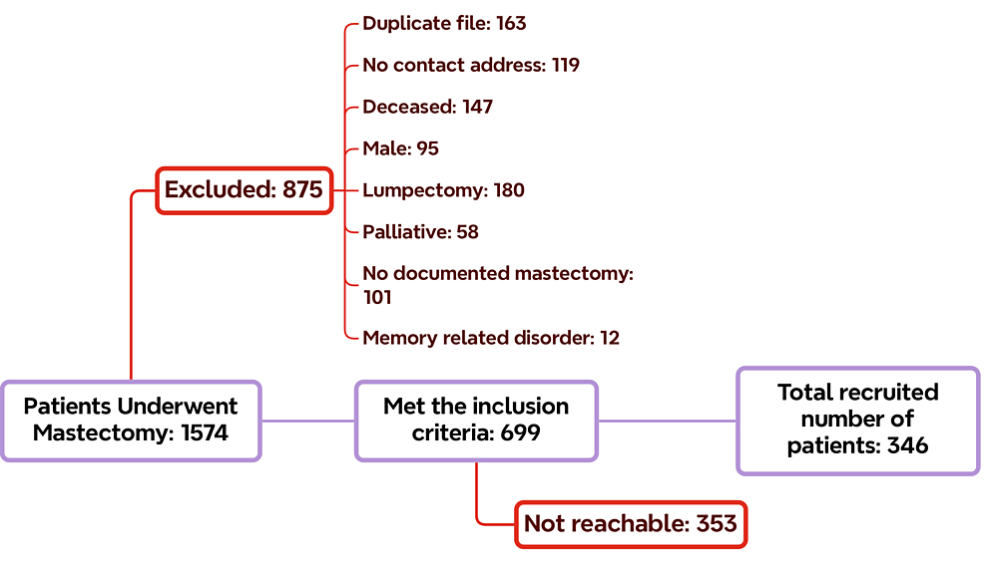
A flowchart of the included and excluded number of patients according to the exclusion criteria

To reduce recall bias, we excluded patients who did not speak Arabic, were illiterate, or had age-related memory disorders. Additionally, family or friends were not eligible to participate on behalf of the patient herself. Our exclusion criteria were patients who only underwent partial mastectomies, lumpectomies, palliative mastectomies, and patients with stage 4 cancer and planned for palliative care, in addition to patients who are male and female patients who passed away (Figure 1).

The objective of this paper is to assess the level of patient satisfaction following mastectomy and cancer treatments, with a specific focus on female patients. We did exclude all male participants because male breast cancer may require different surgical approaches, such as mastectomy, due to the smaller size of the breast tissue. Additionally, male breast cancer patients may not respond as well to hormonal therapies, which are often used to treat female breast cancer patients [10].

We contacted all 699 participants who met our inclusion criteria; however, not all of them responded or answered the phone, so there were multiple attempts to reach them. However, it was agreed to exclude them after three attempts on separate occasions. Moreover, there were patients who already passed away and were not documented in the BestCare system by the time we reached them, and so they were excluded as well. All of them were referred to as “Not reachable.”

Primary and secondary data were used in this study. The first part of the questionnaire was collected from pre-existing information on the online hospital system (BestCare system). The second part was a phone-call-based survey. The collected data were plotted into Google Forms and then transferred to an Excel spreadsheet. The data were also validated whether it could be used for data analysis or not. As such, incomplete or wrongly inserted information of any patient was taken out if otherwise cannot be retained again.

Statistical analysis

The assessed variables were analyzed using Statistical Product and Service Solutions (SPSS) v.26.0 (IBM Corp., Armonk, NY). Continuous variables are shown as mean ± standard deviation (SD) and categorical variables as a number (percentage).

Ethical approval

All procedures were approved by the King Abdullah International Medical Research Center (KAIMARC) (IRB Approval Number IRB/0165/22) and the ethical guidelines of KAIMARC were followed closely. Names of study subjects were not recorded. Informed consent was obtained from all subjects.

## Results

A total of 346 women who survived BC and were 28 years of age or older took part in this study. Of this population, 36.13% were <59 years of age at the time of contact, with a current age mean ± SD = 52.3 ± 11.5. Additionally, 81.5% of participants were married, 49.42% were staged 2, 32.66% were staged 3, 4.34% were staged 1, 93.64% underwent unilateral mastectomy, 85.54% received chemotherapy, 35.54% received radiotherapy, 31.21% hormonal therapy, and 12.72% underwent breast reconstruction.

We documented the stage at the time of the diagnosis to assess the progression of the disease and its effect on patients’ QOL (Table 1). Patients with stage 4 cancer usually undergo palliative care, as well as have a lower baseline of QOL than those with a less progressive disease [11]. However, one patient with stage 4 BC was included because she received treatment with the intention to cure, not to palliate, and this patient's QOL was similar to those of patients with earlier-stage disease.

**Table 1:** The demographics of this study with the stage of cancer and treatment plan

Variable	Value	Percentage (%)
N	346	100%
Age at time of contact		
Age ≤50 years	139	40.17%
Age >50 years	207	59.83%
Marital status		
Married	282	81.50%
Divorced/ separated	29	8.38%
Single	27	7.80%
Cancer stage at time of diagnosis		
Stage 1	15	4.34%
Stage 2	171	49.42%
Stage 3	113	32.66%
Stage 4	1	0.29%
Unknown	47	13.58%
Treatment Plan		
Mastectomy (unilateral)	321	93.64%
Mastectomy (bilateral)	22	6.36%
Neoadjuvant Chemotherapy	149	43.06%
Neoadjuvant Radiotherapy	7	2.02%
Neoadjuvant Hormonal	9	2.60%
Adjuvant Chemotherapy	145	41.91%
Adjuvant Radiotherapy	116	33.53%
Adjuvant Hormonal	98	28.32%
Breast Reconstruction surgery	44	12.72%

The criteria for assigning patient stage as (unknown) were participants whose stage diagnosis was unclear or not found in the patient file, which included 47 (13.58%) participants.

Out of 346 participants interviewed in this study, 86.99% of them reported being satisfied when directly questioned “Are you satisfied with surgery outcomes?” and 68.79% reported being socially unaffected by surgery outcomes when directly questioned “Have you been affected socially because of surgery outcomes?” (Table 2).

**Table 2:** Number of patients with a state of satisfaction after mastectomy and its effect on social life

Variable	Yes (n %)	No (n %)
Satisfaction (n=346)	301 (86.99%)	45 (13.01%)
Effect on social life (n=346)	108 (31.21%)	238 (68.79%)

A total of 301 participants reported being satisfied. According to 78.41% of them, it was due to their faith and acceptance of the disease, 66.45% due to being satisfied with surgery outcomes, 59.8% due to their trust in the surgeon, and 48.84% due to their trust in the institution (Table 3).

**Table 3:** Reasons for satisfaction and dissatisfaction after mastectomy

Data	Patient number			Reasons of satisfaction				Reasons of dissatisfaction								
	Total number of patients	Patients reported satisfaction	Patients reported not satisfaction	Acceptance of disease	satisfied of surgery	satisfied with surgeon	satisfied with the hospital	Body image issues	Psychological effects after organ removal	Redundant skin	Ugly Scar	Lymphedema	Chronic surgical site pain	Post-op complications	Metastasis	Others
Value	346	301	45	236	200	180	147	26	24	13	12	10	5	3	1	7
Percentage %	100%	86.99%	13.01%	78.41%	66.45%	59.80%	48.84%	57.78%	53.33%	28.89%	26.67%	22.22%	11.11%	6.67%	2.22%	15.56%

On the other hand, 45 participants reported being dissatisfied after mastectomy. The reasons for that were 57.78% due to body image issues, 53.33% due to a psychological effect after losing an organ, 28.89% redundant skin, 26.67% ugly scar, 22.22% lymphedema, 11.11% chronic surgical site pain, 6.67% post ope complications, 2.22% metastasis, and 15.56% reported unspecified reasons for their dissatisfaction listed as "Other" (Table 3).

A total of 108 participants had their social life impacted. The highest effect accounted for 70.37% was loss of confidence, followed by 56.48% social withdrawal, 39.81% embarrassment, and 17.59% inability to obtain external breast prostheses. The lowest reasons are listed in Table 4.

**Table 4:** Effects on patients' social life after mastectomy

Variable	Value	Percentage (%)
Patients reported being socially affected	108	31.21%
Effect on social life		
Loss of confidence	76	70.37%
Social withdrawal	61	56.48%
Embarrassment	43	39.81%
No breast prosthesis	19	17.59%
Occupational impact	4	3.70%
Limited clothing	3	2.78%
Other	4	3.70%

There were four participants who reported individually distinct reasons: 3.7% were listed as "Other," which subdivides into 0.93% expensive reconstruction surgery costs, 0.93% had no proper psychological support, 0.93% attempted suicide, and 0.93% unspecified reason (Table 4).

We listed all neoadjuvant and adjuvant treatment options that patients received besides mastectomy and the satisfaction state of each one (Table 5).

**Table 5:** The number of satisfied vs. not satisfied patients with each treatment plan

Treatment plan	n	Satisfied	Not satisfied
Mastectomy (Unilateral)	324	280 (86.42%)	41 (12.65%)
Mastectomy (Bilateral)	22	19 (86.36%)	3 (13.64%)
Neoadjuvant Chemotherapy	149	131 (87.92%)	18 (12.08%)
Neoadjuvant Radiotherapy	7	7 (100%)	0 (0%)
Neoadjuvant Hormonal	9	9 (100%)	0 (0%)
Adjuvant Chemotherapy	145	122 (84.14%)	23 (15.86%)
Adjuvant Radiotherapy	116	103 (88.79%)	13 (11.21%)
Adjuvant Hormonal	98	83 (84.69%)	15 (15.31%)
Reconstruction	44	35 (79.55%)	9 (20.45%)

## Discussion

While the majority of participants in the study reported being satisfied with the outcome after mastectomy, it is important to recognize that many of them still struggled with a range of psychological, social, and emotional challenges during their treatment [5,6]. Mastectomy is a major surgery that can overwhelmingly impact patients' lives [6,7]. This kind of distress can result in depressive symptoms among patients [8]. As we have observed, losing an organ is a devastating experience for patients both emotionally and physically, affecting their overall QOL [7-9].

On the other hand, a small percentage of patients were not satisfied after mastectomy. Most of them have attributed their dissatisfaction to a wide range of psychological issues arising from the recency of mastectomy [9]. The nature of this surgery involves the removal of one or both breasts, which can be a traumatic experience in terms of body image and sense of self [11,12]. This can lead to a range of psychological issues, including depression, anxiety, loss of self-worth, and a drop in the overall QOL [12].

Moreover, in the Saudi community, social support is provided by family, friends, and society [12]. As shown in Table 2, most participants did not struggle socially throughout their treatment plan. A supportive circle of friends and family can help lift some of the disease burden when the patient receives empathy and support [12,13], resulting in higher contentment and better treatment adherence by the patient [12]. Studies emphasized the role of family and societal support in reaching better outcomes, especially in cancer patients [12,13]. Conversely, other participants reported struggling with stigmatization and discrimination for their diagnosis, as shown in Table 4. Those participants struggled to maintain their supporting networks and were intolerant to dealing with social connections, leading to social withdrawal from family, friends, and social gatherings [13]. Maladaptive handling of psychological distress can develop other complications throughout the treatment of cancer [14].

On the emotional level, patients have experienced different impactful emotions described as feelings of hopelessness, overwhelm, stress, and exhaustion. To address these challenges, healthcare professionals must take a holistic approach to patient care, addressing both the physical and psychological needs of the patients. Providing proper psychological support and resources will yield higher patient satisfaction and improvement in their QOL [15].

Reasons for satisfaction

According to the principle of accepting destiny in the Islamic community, individuals shall accept both good and bad events that may occur in their lives, including illnesses [15]. The respective patients in this study were asked about the reason for their satisfaction state, and many of them attributed it to their acceptance of their faith (Table 6). For those patients, their religious beliefs provided them with a sense of comfort and acceptance, allowing them to cope with their challenges [15]. Another reason to be satisfied with undergoing mastectomy is the overall outcome. For many patients, surgery is considered the definitive cancer management, and it represents a significant milestone in their journey toward recovery [16]. Moreover, they expressed feelings of relief and closure as it symbolized a sense of victory against cancer and a renewed sense of hope and optimism [16,17].

**Table 6:** Treatment options and reported reasons of satisfaction

Treatment Plan	Reasons reported for being satisfied			
	Acceptance of disease (n)	satisfied of surgery (n)	satisfied with surgeon (n)	satisfied with the hospital (n)
Mastectomy (unilateral)	224	186	169	139
Mastectomy (bilateral)	12	12	8	6
Neoadjuvant Chemotherapy	99	91	76	61
Neoadjuvant Radiotherapy	7	5	4	2
Neoadjuvant Hormonal	8	5	4	4
Adjuvant Chemotherapy	99	77	69	48
Adjuvant Radiotherapy	81	66	55	45
Adjuvant Hormonal	63	50	44	41
Reconstruction	24	27	24	21

This high level of satisfaction can be attributed to the fact that all patients receive a tailored treatment plan to their specific needs and circumstances, which is consistent with current best practices in cancer care [18]. As shown in the literature, an individually tailored and multidisciplinary-team approach to patients shows better outcomes [18]. In addition, patients attributed their satisfaction to the surgeon's efforts to be supportive by building a good rapport, providing empathy, preserving autonomy, and thoroughly explaining the surgical plan [19,20]. Moreover, the ability of the surgeon to explain the surgical plan in detail, including the different surgical approaches and alternatives, discuss the need and the timing of the surgery, and outline the risks and benefits of the surgery in order to establish a treatment plan [20].

Healthcare services are offered to all patients to help in their before and after-surgery stay at the hospital. Patients are given information including what to expect before, during, and after the procedure [20]. They are also given pain management after surgery and physical therapy for those who may need it [21]. Patients who are satisfied with the care they receive are more likely to have a positive experience and faster recovery [22]. They are more likely to be satisfied with their care if the facilities are clean, well-maintained, comfortable, combined with a friendly, helpful, and knowledgeable staff [23]. By providing the aforementioned high-quality healthcare services, hospitals can improve patients’ satisfaction and help patients achieve better outcomes [23].

Reasons for dissatisfaction

It is important to note that not all patients feel the same sense of satisfaction or acceptance with their illness or treatment. For some patients, the physical and emotional challenges of BC and its treatment may be overwhelming and may require additional support and resources to cope with (Table 7).

**Table 7:** Treatment options and reported reasons for dissatisfaction

Treatment plan	Reasons reported for being not satisfied						
	Psychological effect (n)	Redundant skin (n)	Body image issues (n)	Lymphedema (n)	Ugly scar (n)	Metastasis (n)	Chronic surgical site pain (n)
Mastectomy (unilateral)	23	11	24	10	11	0	2
Mastectomy (bilateral)	1	2	2	0	1	1	0
Neoadjuvant chemotherapy	10	6	10	4	3	1	2
Neoadjuvant radiotherapy	0	0	0	0	0	0	0
Neoadjuvant hormonal	0	0	0	0	0	0	0
Adjuvant chemotherapy	12	8	11	6	6	0	0
Adjuvant radiotherapy	6	4	7	3	0	0	0
Adjuvant hormonal	5	3	6	4	4	1	1
Reconstruction	7	4	6	4	3	0	0

Following a comprehensive analysis of this study subjects responses, the authors identified several key factors that contributed to patients’ dissatisfaction, which can be broadly categorized into four main areas: psychological effects, postoperative complications, and body image issues. This categorization was created according to the most reported causes by our sample of patients.

Psychological effects, first of all, from losing an organ can significantly impact a person [6,7]. Breast removal, in particular, threatens a woman's identity and challenges her self-integrity because losing a part seen as a symbol of muliebrity can lead to changes in the sense of femininity of women [7,8]. Even after successful treatment, patients still feel unsafe and afraid of the disease’s recurrence [8,9]. It can be extremely difficult for women to adapt to their new image, as they may feel that their sense of self-worth has been fundamentally altered after surgery [9]. Not only that but also their spouses may look at them differently. This is because surgeries for BC will lead to changes in the outer appearance of the body whether total loss of the breast, changes in size and shape of the breast, presence of scars, and presence of redundant skin [9,17]. The journey of BC from diagnosis to the end of treatment can affect spousal relationships [11,13], to the extent that four married women reported being divorced because of their spouse's inability to accept their new body. Moreover, 38 (11.10%) patients reported refraining from their partner from intimacy and social support. It is a huge burden for such patients adjusting to their new challenges with minimal or no social support [14]. Those patients suffered from low self-esteem, loss of confidence, and loss of partner support all at once (Table 7).

Mastectomy carries both benefits and risks of complication, as any other surgery [20]. One complication is chronic surgical site pain, which has been reported by patients as disturbing their daily living [24]. Such complications are usually due to nerve injury, infection, scar tissue formation, and structural damage at the excision site [24,25]. Pain, in general, can prevent people from engaging in activities, which also affects the level of productivity of a person in their work or education [25]. Another recognized risk is lymphedema, which is a condition characterized by the accumulation of lymphatic fluid in the interstitial tissues after axillary lymph node dissection, a technique indicated during a mastectomy surgery, manifested by swelling of the hand, arm, breast, or trunk [26]. This complication, if occurred, can be challenging and lead to decreased QOL because of body changes, pain in the affected area, and a possible alteration of arm functionality [26].

Effects on the social life

This study identified the most common reasons for social impact on women following mastectomy. The most commonly reported reasons for social impact on women after undergoing mastectomy in our paper are loss of confidence, inability to attend family or social gatherings, embarrassment, withdrawal, inability to obtain breast prosthesis, affected school/work due to prolonged absenteeism, and limited clothing.

Many patients experience a drop in self-esteem, so they begin to withdraw from social activities due to fear of judgment and disease stigma [27,28]. Patients may feel self-conscious or embarrassed about their appearance and may be hesitant to engage in social activities as a result [29,30]. This can lead to feelings of isolation and loneliness, which can further exacerbate the psychological impacts after surgery [29].

Many patients experienced changes in their self-identity and self-image after breast-removing surgery [30,31]. According to our patients, they had to deal with new bodies and adapt to newer ways to dress. Additionally, the costs of cosmetic breast reconstruction surgery were prohibitively unaffordable to them. Therefore, a more affordable alternative is an external breast prosthesis, an artificial breast-shape material that is inserted into the bra to replace the lost breast [32]. External breast prostheses can be a viable option for some patients, but they do have some limitations; for instance, they may not provide the same level of comfort or natural appearance when compared to cosmetic breast reconstruction [32]. They may require regular adjustments or replacements over time or cause discomfort or skin irritation from wearing them [32]. It was noticed that there were barriers to obtaining these breast prostheses even if patients could afford them, such as product availability and limited providers, as shown in Table 4. Therefore, many patients reported having issues with clothes in general, forcing them to be extremely limited to specific clothing options.

Furthermore, patients experienced feelings of fear of recurrence, which led to anxiety and stress [33]. This fear may begin with the completion of treatment and could continue over the years, causing a significant impact on patients' QOL [33]. Other patients reported that they had problems in their careers or education. According to them, this was due to treatment appointments interfering with the timeline of their schedules. They were absent from work or school to receive their treatment at the hospital. After mastectomy, they faced prolonged recovery time, which led to further absenteeism. This can be attributed to a few factors, including surgery site pain, fatiguability, and postoperative complications requiring more hospital visits [34].

This study also observed less common, yet severe, factors that may contribute to psychological distress following breast removal surgery. For example, a patient who underwent a mastectomy followed by cosmetic breast reconstruction surgery attempted suicide due to dissatisfaction with the surgical outcome, which had a profound impact on her psychological well-being, as the breasts are an integral part of her body image. Fortunately, the patient was rescued in the emergency department, and her life was preserved. This raises the importance of suicidal ideation screening among patients undergoing treatment for BC.

Limitation and recommendation

The study had a few limitations, largely due to inaccessible patients or incomplete information. A total of 147 patients passed away, and 353 patients were not reachable. Moreover, patients before the year 2016, before the integration of the electronic file system in our hospital, had obsolete contact information, in addition to the inaccuracy in presenting the patient’s case information such as types of therapy. 

It is also important to mention that, while interviewing patients, most of them, whether satisfied or unsatisfied, attributed their level of satisfaction to more than one reasons/causes. This was the main reason that we were unable to identify a single reason for patients' satisfaction status. However, we were able to present these most reported reasons as numbers and percentages.

We highly recommend looking into patients diagnosed and treated for BC, specifically patients who were satisfied, to assess whether or not their coping mechanisms were effective and resulting in their satisfaction. As patients were from a variety of age groups, one must ask whether they were unsatisfied at baseline and then developed satisfaction over time. Further research is needed to confirm these findings and to identify other sources that may influence patient satisfaction following mastectomy and its associated treatments. Such research could help in the development and advancement of the quality of care provided to patients.

## Conclusions

The study findings highlight the significant impact of mastectomy on patients' lives, whether or not they are satisfied. It is crucial for physicians to be aware of such challenges to screen for depression, anxiety, and suicidal ideation. This could be a highly valuable step to increase patients' QOL and satisfaction after mastectomy. This highlights the importance of considering individual patient experiences and circumstances when evaluating treatment outcomes and patient satisfaction. Further research is needed to confirm these findings and to identify other sources and factors that may influence patient satisfaction following mastectomy and its associated treatments. Such research could help in the development and advancement of the quality of care provided to patients.

## Figures and Tables

**Figure 2:**
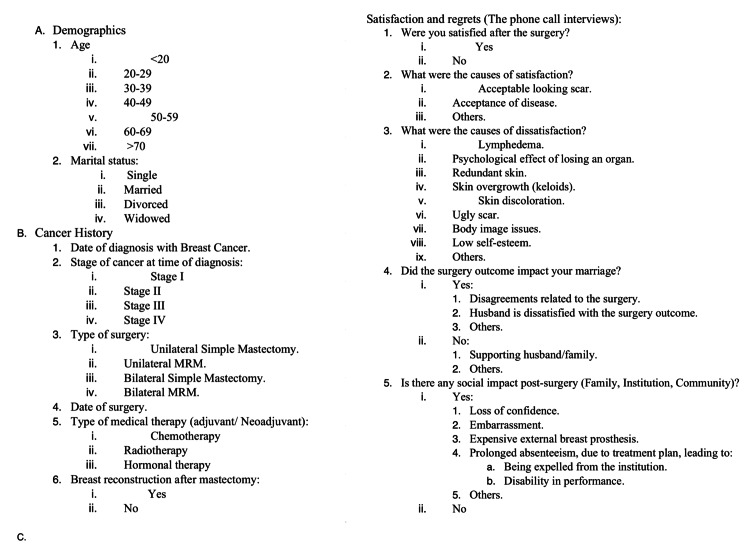
Patients' demographics and phone call interview script
